# Comparison of rivaroxaban and low molecular weight heparin in the treatment of cancer-associated venous thromboembolism: a Swedish national population-based register study

**DOI:** 10.1007/s11239-024-02992-1

**Published:** 2024-05-12

**Authors:** Marie Linder, Anders Ekbom, Gunnar Brobert, Kai Vogtländer, Yanina Balabanova, Cecilia Becattini, Marc Carrier, Alexander T. Cohen, Craig I. Coleman, Alok A. Khorana, Agnes Y. Y. Lee, George Psaroudakis, Khaled Abdelgawwad, Marcela Rivera, Bernhard Schaefer, Diego Hernan Giunta

**Affiliations:** 1 Department of Medicine Solna, Clinical Epidemiology/ Centre for Pharmacoepidemiology, Karolinska Institutet, Karolinska University Hospital, Stockholm, Sweden; 2 Bayer AG, Berlin, Germany; 3 Department of Internal and Emergency Medicine, Stroke Unit, University of Perugia, Perugia, Italy; 4 Department of Medicine, Ottawa Hospital Research Institute at the University of Ottawa, Ottawa, Canada; 5 Department of Haematological Medicine, Guy’s and St Thomas’ NHS Foundation Trust, King’s College London, London, UK; 6 School of Pharmacy, University of Connecticut, Storrs, CT USA; 7 Cleveland Clinic and Case Comprehensive Cancer Center, Cleveland, OH USA; 8 University of British Columbia and BC Cancer, Vancouver, Canada; 9 Consultant for Bayer AG, Berlin, Germany; 10 Present Address: Janssen Research and Development, Barcelona, Spain

**Keywords:** Cancer-associated thrombosis, Recurrent venous thromboembolism, Major bleeding, Rivaroxaban, Low molecular weight heparin

## Abstract

**Background:**

Treating cancer-associated venous thromboembolism (CAT) with anticoagulation prevents recurrent venous thromboembolism (rVTE), but increases bleeding risk.

**Objectives:**

To compare incidence of rVTE, major bleeding, and all-cause mortality for rivaroxaban versus low molecular weight heparin (LMWH) in patients with CAT.

**Methods:**

We developed a cohort study using Swedish national registers 2013–2019. Patients with CAT (venous thromboembolism within 6 months of cancer diagnosis) were included. Those with other indications or with high bleeding risk cancers were excluded (according to guidelines). Follow-up was from index-CAT until outcome, death, emigration, or end of study. Incidence rates (IR) per 1000 person-years with 95% confidence interval (CI) and propensity score overlap-weighted hazard ratios (HRs) for rivaroxaban versus LMWH were estimated.

**Results:**

We included 283 patients on rivaroxaban and 5181 on LMWH. The IR for rVTE was 68.7 (95% CI 40.0–109.9) for rivaroxaban, compared with 91.6 (95% CI 81.9–102.0) for LMWH, with adjusted HR 0.77 (95% CI 0.43–1.35). The IR for major bleeding was 23.5 (95% CI 8.6–51.1) for rivaroxaban versus 49.2 (95% CI 42.3–56.9) for LMWH, with adjusted HR 0.62 (95% CI 0.26–1.49). The IR for all-cause mortality was 146.8 (95% CI 103.9–201.5) for rivaroxaban and 565.6 (95% CI 541.8–590.2) for LMWH with adjusted HR 0.48 (95% CI 0.34–0.67).

**Conclusions:**

Rivaroxaban performed similarly to LMWH for patients with CAT for rVTE and major bleeding. An all-cause mortality benefit was observed for rivaroxaban which potentially may be attributed to residual confounding.

**Trial registration number:**

NCT05150938 (Registered 9 December 2021).

**Supplementary Information:**

The online version contains supplementary material available at 10.1007/s11239-024-02992-1.

## Highlights

Treating cancer-associated venous thromboembolism with anticoagulation prevents recurrent VTE, but may increase the risk of bleeding. DOACs have easier administration and proven higher treatment adherence compared to LMWH.

Excluding cancer with high risk of bleeding, rivaroxaban performed similarly to LMWH for patients with cancer and VTE for recurrent VTE and major bleeding.

An all-cause mortality benefit was observed for rivaroxaban compared to LMWH which potentially may be attributed to residual confounding.

**Supplementary Information:**

The online version contains supplementary material available at 10.1007/s11239-024-02992-1.

## Introduction

Cancer-associated thrombosis (CAT) is a serious complication and a leading cause of death second to cancer progression among patients with cancer [1–3]. Venous thromboembolism (VTE) includes deep vein thrombosis (DVT) and pulmonary embolism (PE). Whether or not the long-term mortality is increased in individuals surviving the initial VTE episode is less clear [4]. Risk of VTE is 4- to 7-fold higher in patients with cancer compared with the general population [5], with incidence rates (IRs) for all cancer types ranging from 2 to 12 cases per 100 person-years [6]. The risk of recurrent VTE (rVTE) events is also increased in individuals with anticoagulants and cancer-specific treatments [6].

Recommended treatments for CAT have changed recently, now including direct oral anticoagulants (DOACs; e.g. rivaroxaban, edoxaban, apixaban) and low molecular weight heparin (LMWH; e.g. dalteparin, enoxaparin, tinzaparin) as standard of care for CAT in patients with low risk of gastrointestinal or urogenital bleeding [7–9]. Although meta-analyses have confirmed the role of LMWH in both the initial anticoagulation period and for long-term treatment, patients’ adherence was low in long-term treatment studies given the requirements for daily subcutaneous injection [10]. In contrast, the convenience of DOAC may improve adherence and patient outcomes [11]. Shared decision-making with patients is recommended, considering the potential lower risk of VTE recurrences associated with DOACs but higher bleeding risk as compared to LMWH [8, 9]. Rivaroxaban may be more effective in treating patients with CAT, significantly reducing recurrent thrombosis compared with LMWH, without increasing major bleeding and all-cause mortality, but there is heterogeneity among published studies [12–18].

The present Observational Study of Cancer Associated Thrombosis for Rivaroxaban in SwEden (OSCAR-SE) aimed at examining the incidence of rVTE, major bleeding, and all-cause mortality in patients diagnosed with CAT treated with rivaroxaban compared with LMWH, based on data from national health registries in Sweden.

## Methods

### Ethical approval

An ethical application was submitted to the national ethical committee. Similarly, a scientific application was submitted to the national board of health and welfare asking for permission to obtain the necessary record linkages and release of data from named sources. All analyses were conducted on pseudo-anonymized individual data.

### Study design

This cohort study was based on available nationwide health register data in Sweden. All individuals with a Swedish personal identification number and a diagnosis of cancer between 2013 and 2019 followed by a subsequent diagnosis of VTE within the next 6 months were identified and followed until the date of outcome, death, emigration, or end of follow-up on 31 December, 2020, whichever occurred first. The date of the first VTE after cancer diagnosis was the index event corresponding to start of follow-up.

### Setting

All residents in Sweden diagnosed with cancer during the study period were identified through the Swedish Cancer Register (SCR) and linked to other national health and sociodemographic registers (National Patient Register [NPR], Prescribed Drug Register [PDR], Total Population Register [TPR], and Cause of Death Register [CDR]). Individuals were linked through their unique personal identifier given at birth or immigration.

Patients included fulfilled the inclusion criteria of having a non-autopsy cancer recorded in SCR 2013–2019 with a subsequent VTE diagnosis registered in NPR (inpatient or outpatient) in the following 183 days, living in Sweden at least 183 days before the index-VTE and being 18 years of age or older at the index-VTE. Moreover, they fulfilled none of the exclusion criteria of excluding diagnoses (atrial fibrillation, hip/knee replacement, or acute coronary syndrome) or treatment (DOAC, vitamin K antagonist, or LMWH), both within 183 days before the index-VTE, and had not a cancer type associated with increased bleeding risk according to the International Society on Thrombosis and Haemostasis (ISTH) guideline [9]. Excluded cancers were lip/upper gastrointestinal cancer, malignant immunoproliferative diseases, leukaemia and non-melanoma skin cancer (Supplementary Table S1).

### Data sources

The data quality in SCR is high, with almost 99% of all cancer diagnoses morphologically verified, and with quality controlled at regional cancer centres before submission to the National Board of Health and Welfare [19]. The NPR, used for outcomes and comorbidities, includes information about diagnoses and surgical procedures from hospitals and visits to specialist care. Diagnoses are coded according to the current 10th version of International Classification of Diseases (ICD-10). The validity of NPR is high, with positive predictive values generally around 85–95% for most diagnoses [20]. The PDR provides information on all dispensed prescriptions from pharmacies with substances coded according to the Anatomic Therapeutic Chemical Classification System, date of purchase, and amount dispensed in defined daily doses [21]. The TPR holds information about, among others, education, employment status, income, marital status, region of residency, and migration [22]. The CDR provides information about causes and dates of death [23].

### Outcomes variables

The outcome rVTE was defined as a diagnosis of DVT or PE recorded as main diagnosis at discharge from hospital. Major bleeding was defined as a diagnosis of intracranial, gastrointestinal, urogenital, or other bleeding recorded as a main diagnosis at discharge from hospital. All-cause mortality was retrieved from CDR. For codes defining outcomes, see Supplementary Table S1.

### Statistical methods

Exposure was defined as the first dispensation of rivaroxaban or LMWH within 28 days after the index VTE. The main exposure measure was the intention-to-treat (ITT) approach, i.e., each individual was assigned to their first recorded treatment after their index-VTE and was assumed to stay on that until censoring. As a sensitivity analysis, the on-treatment exposure measure was applied, where each individual’s exposure stopped at estimated end of supply, or switch. Exposure duration was estimated according to consecutive dispensed prescriptions and amount of dispensed medication.

IRs with 95% Poisson confidence intervals (CIs) were calculated overall and for 3, 6, 12, and 24 months after index CAT.

The comparisons of the independent outcomes rVTE, major bleeding, and all-cause mortality used propensity score (PS) overlap weights [24]. Overlap weighting assigns weights to each patient that are proportional to the probability of belonging to the opposite treatment group. The PS model included 85 variables identified as potential confounders, including demographics, comorbidities, medications, and cancer characteristics, see Supplementary Table S2.

Cox proportional hazards regression were applied to compare time to event for the outcomes between treatment groups using the robust variance sandwich estimator [25]. The only independent variable included in the Cox models was anticoagulant received (rivaroxaban or LMWH), since the PS overlap weights balance the characteristics included in the PS. Standardized differences before/after adjusting by PS overlap weights were explored using Love plots. Hazard ratios (HRs) are presented with 95% CIs.

As a sensitivity analysis, death was considered a competing risk for the outcomes rVTE and major bleeding. Fine–Gray regression subhazards [26] were estimated using PS overlap weighting. As an additional sensitivity analysis, we compared all DOACs (rivaroxaban, dabigatran, apixaban, edoxaban) to LMWH (Supplementary Table S5).

## Results

**Fig. 1:**
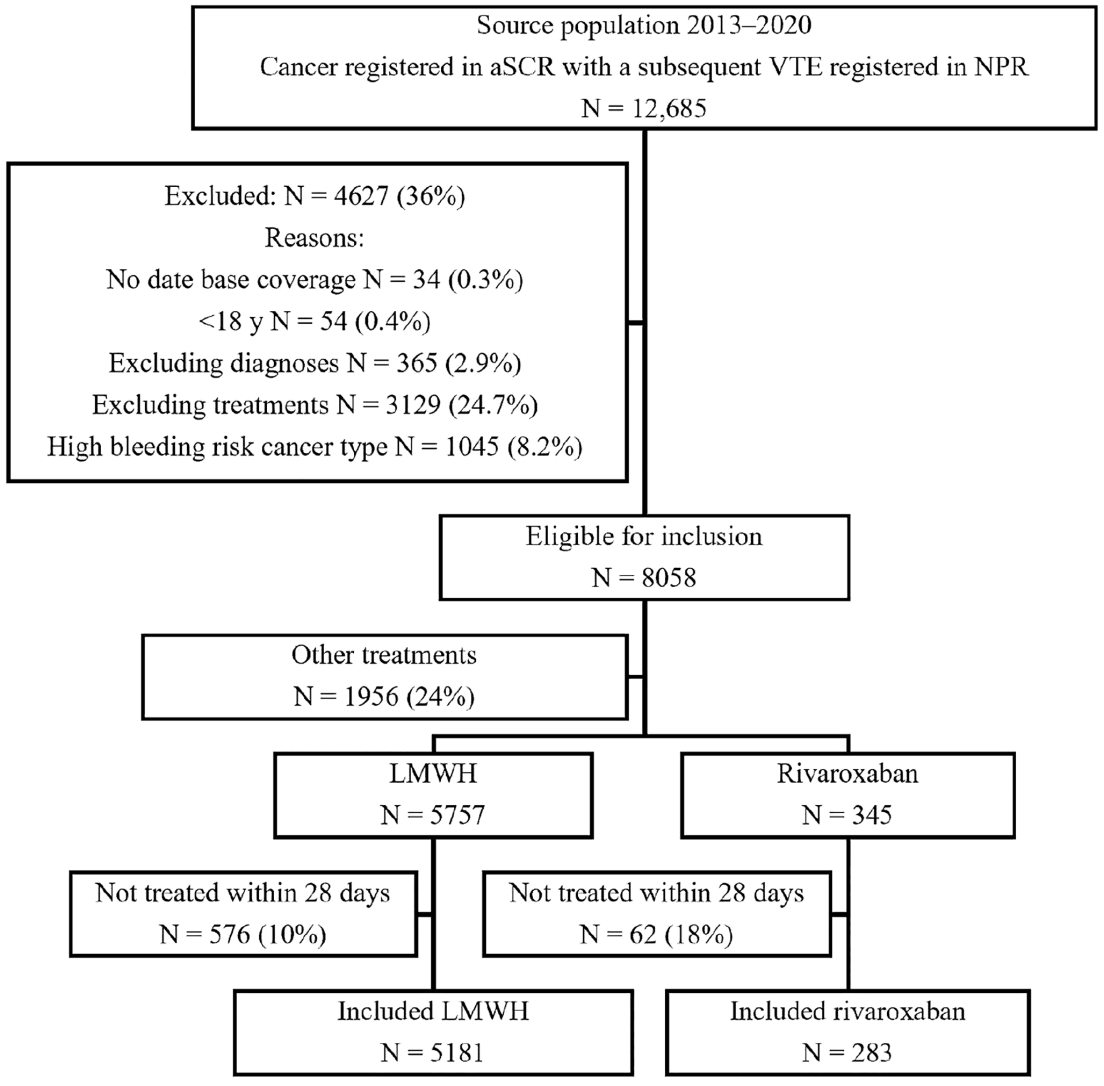
Inclusion flowchart from source population of individuals with cancer induced thrombosis to study population of individuals treated with rivaroxaban or LMWH

A total of 5,464 individuals with CAT were included, of whom 283 used rivaroxaban and 5,181 used LMWH (Fig. 1). The proportion of included patients varied over study years, with more rivaroxaban-treated patients included later in the study period, whereas the inclusion of LMWH users was relatively constant over time. The majority of index-VTEs were PE, 55% for LMWH and 60% for rivaroxaban. Baseline characteristics are shown in Table 1. After applying PS overlap weighting, all included baseline characteristics were well balanced (see Table 1, Supplementary Table S2, and Supplementary Fig. S1).

**Table 1:** Selected baseline characteristics after exclusion on treatment, including ISTH cancers only, with treatment within 28 days after index-VTE; frequency (proportion) for rivaroxaban and LMWH, before and after PS overlap weighting

		Before PS overlap weighting		After PS overlap weighting	
Variable	Value	Rivaroxaban	LMWH	Rivaroxaban	LMWH
Total number		283	5181	200	200
Age	<65	78 (28%)	1739 (34%)	57 (28%)	57 (28%)
	>=65	205 (72%)	3442 (66%)	143 (72%)	143 (72%)
Sex	Female	137 (48%)	2952 (57%)	103 (51%)	103 (51%)
	Male	146 (52%)	2229 (43%)	97 (49%)	97 (49%)
Inclusion year	2013	12 (4%)	766 (15%)	7 (3%)	7 (3%)
	2014	40 (14%)	744 (14%)	22 (11%)	22 (11%)
	2015	49 (17%)	746 (14%)	36 (18%)	36 (18%)
	2016	37 (13%)	753 (15%)	32 (16%)	32 (16%)
	2017	56 (20%)	746 (14%)	33 (17%)	33 (17%)
	2018	50 (18%)	769 (15%)	36 (18%)	36 (18%)
	2019	39 (14%)	657 (13%)	34 (17%)	34 (17%)
Type of VTE	DVT	102 (36%)	2122 (41%)	73 (36%)	73 (36%)
	PE	171 (60%)	2828 (55%)	120 (60%)	120 (60%)
	Both	10 (4%)	231 (4%)	7 (3%)	7 (3%)
Cancer type	Oral cavity and pharynx	2 (0.7%)	67 (1%)	2 (1%)	2 (1%)
	Digestive organs	52 (18%)	1561 (30%)	42 (21%)	42 (21%)
	Respiratory and intrathoracic organs	14 (5%)	946 (18%)	12 (6%)	12 (6%)
	Bone and articular cartilage	0 (0.00%)	11 (0.2%)	0 (0.00%)	0 (0.00%)
	Malignant melanoma	37 (13%)	51 (1%)	15 (8%)	15 (8%)
	Mesothelial and soft tissue	2 (0.7%)	57 (1%)	2 (0.9%)	2 (0.9%)
	Breast	30 (11%)	606 (12%)	24 (12%)	24 (12%)
	Female genital organs	14 (5%)	382 (7%)	12 (6%)	12 (6%)
	Male genital organs	58 (20%)	265 (5%)	33 (17%)	33 (17%)
	Urinary tract	17 (6%)	321 (6%)	14 (7%)	14 (7%)
	Eye, brain, and other parts of central nervous system	27 (10%)	350 (7%)	22 (11%)	22 (11%)
	Thyroid and other endocrine glands	11 (4%)	30 (0.6%)	6 (3%)	6 (3%)
	Ill-defined, secondary, and unspecified	1 (0.4%)	188 (4%)	1 (0.5%)	1 (0.5%)
	Lymphoid, haematopoietic and related tissue	18 (6%)	346 (7%)	15 (7%)	15 (7%)
Aggregated TNM	0	30 (11%)	159 (3%)	17 (9%)	17 (9%)
	1	70 (25%)	718 (14%)	44 (22%)	44 (22%)
	2	58 (20%)	820 (16%)	38 (19%)	38 (19%)
	3	26 (9%)	1080 (21%)	22 (11%)	22 (11%)
	4	20 (7%)	914 (18%)	17 (9%)	17 (9%)
	Missing	79 (28%)	1490 (29%)	62 (31%)	62 (31%)
Hospital duration during 1 year before index date	0 weeks	158 (56%)	2443 (47%)	103 (52%)	103 (52%)
	1–2 weeks	84 (30%)	1779 (34%)	65 (33%)	65 (33%)
	3–10 weeks	38 (13%)	904 (17%)	31 (16%)	31 (16%)
	>10 weeks	3 (1%)	55 (1%)	0 (0.2%)	0 (0.2%)
Education	Elementary school	97 (34%)	1484 (29%)	71 (36%)	71 (36%)
	High school	114 (40%)	2307 (45%)	80 (40%)	80 (40%)
	College/university	63 (22%)	1282 (25%)	43 (22%)	43 (22%)
	Postgraduate	8 (3%)	55 (1%)	6 (3%)	6 (3%)
	Missing	1 (0.4%)	53 (1%)	0 (0.00%)	0 (0.00%)
Employment	Employed	87 (31%)	1745 (34%)	64 (32%)	64 (32%)
	Not employed	196 (69%)	3432 (66%)	136 (68%)	136 (68%)
	Missing	0 (0.00%)	4 (0.08%)	0 (0.00%)	0 (0.00%)
Income quintiles	Low	61 (22%)	1109 (21%)	44 (22%)	44 (22%)
	Low–mid	58 (20%)	1055 (20%)	43 (21%)	43 (21%)
	Mid	59 (21%)	1080 (21%)	40 (20%)	40 (20%)
	Mid–high	52 (18%)	985 (19%)	38 (19%)	38 (19%)
	High	53 (19%)	948 (18%)	36 (18%)	36 (18%)
	Missing	0 (0.00%)	4 (0.08%)	0 (0.00%)	0 (0.00%)
Marital status	Married	145 (51%)	2754 (53%)	100 (50%)	100 (50%)
	Divorced	43 (15%)	914 (18%)	31 (16%)	31 (16%)
	Unmarried	45 (16%)	867 (17%)	34 (17%)	34 (17%)
	Widowed	50 (18%)	642 (12%)	35 (17%)	35 (17%)
	Missing	0 (0.00%)	4 (0.08%)	0 (0.00%)	0 (0.00%)
Region	Predominantly urban (Stockholm)	12 (4%)	1002 (19%)	11 (5%)	11 (5%)
	Intermediate (Malmö, Gothenburg)	122 (43%)	1544 (30%)	84 (42%)	84 (42%)
	Predominantly rural (all other)	149 (53%)	2631 (51%)	104 (52%)	104 (52%)
	Missing	0 (0.00%)	4 (0.08%)	0 (0.00%)	0 (0.00%)

ISTH, International Society on Thrombosis and Haemostasis; LMWH, low molecular weight heparin; PE, pulmonary embolism; PS, propensity score; TNM, tumour, nodes and metastases; VTE, venous thromboembolism

**Fig. 2:**
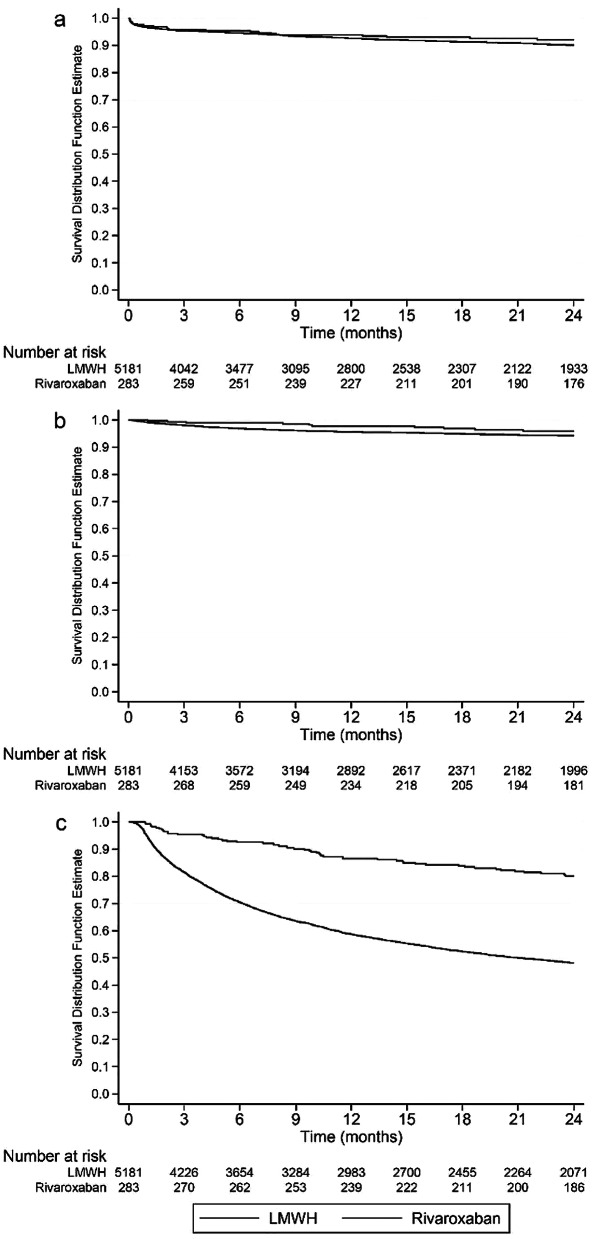
Kaplan–Meier graphs for all outcomes by treatment. Panel (**a**) = Recurrent VTE, panel (**b**) = Major bleeding, and panel (**c**) = All-cause mortality. LMWH, low molecular weight heparin; VTE, venous thromboembolism. Graph created using STATA, StataCorp. 2020. Stata Statistical Software: Version 17.0. College Station, TX: StataCorp LLC

Table 2 includes IRs, weighted and unweighted HRs and sub-HRs for rVTE, major bleeding, and all-cause mortality, comparing rivaroxaban with LMWH under ITT exposure. Kaplan–Meier graphs for all outcomes by treatment are presented in Fig. 2. Comparison of all DOACs with LMWH under ITT exposure showed similar results as presented in Supplementary Table S5.

**Table 2:** Incidence rates, unweighted and weighted hazard ratios from Cox regression and unweighted and weighted subhazards from Fine–Gray regression for recurrent VTE and major bleeding; rivaroxaban vs. LMWH under ITT exposure definition

Outcome	Months follow-up	Group	Events (*N*)	Person-years	IR (CI) per 1000 PY	Unweighted HR (CI)*	Weighted HR (CI)	Unweighted sub-HR (CI)^a^	Weighted sub-HR (CI)
Recurrent VTE	0–3	RVX	12	66	181.1 (93.55–316.3)	0.91 (0.51–1.63)	0.80 (0.40–1.60)	0.94 (0.52–1.68)	0.81 (0.33–2.02)
		LMWH	230	1121	205.2 (179.5–233.5)	1 (Ref)	1 (Ref)	1 (Ref)	1 (Ref)
	0–6	RVX	13	129	100.5 (53.53–171.9)	0.84 (0.48–1.46)	0.75 (0.38–1.45)	0.89 (0.51–1.56)	0.77 (0.32–1.83)
		LMWH	262	2043	128.3 (113.2–144.8)	1 (Ref)	1 (Ref)	1 (Ref)	1 (Ref)
	0–12	RVX	17	248	68.66 (40.00–109.9)	0.81 (0.50–1.32)	0.77 (0.43–1.35)	0.93 (0.57–1.51)	0.81 (0.38–1.74)
		LMWH	328	3583	91.55 (81.91–102.0)	1 (Ref)	1 (Ref)	1 (Ref)	1 (Ref)
	0–24	RVX	21	447	47.00 (29.09–71.84)	0.78 (0.50–1.21)	0.73 (0.44–1.23)	0.96 (0.62–1.48)	0.80 (0.40–1.61)
		LMWH	393	5896	66.66 (60.23–73.59)	1 (Ref)	1 (Ref)	1 (Ref)	1 (Ref)
	Overall	RVX	25	850	29.41 (19.03–43.41)	0.73 (0.49–1.10)	0.70 (0.44–1.13)	0.98 (0.65–1.46)	0.80 (0.42–1.53)
		LMWH	473	10,524	44.95 (40.99–49.19)	1 (Ref)	1 (Ref)	1 (Ref)	1 (Ref)
Major bleeding	0–3	RVX	2	68	29.39 (3.56–106.2)	0.35 (0.09–1.40)	0.40 (0.09–1.74)	0.37 (0.09–1.51)	0.41 (0.06–2.92)
		LMWH	93	1150	80.87 (65.27–99.07)	1 (Ref)	1 (Ref)	1 (Ref)	1 (Ref)
	0–6	RVX	3	133	22.55 (4.65–65.90)	0.32 (0.10–0.99)	0.42 (0.12–1.38)	0.36 (0.12–1.14)	0.44 (0.09–2.16)
		LMWH	141	2096	67.26 (56.62–79.32)	1 (Ref)	1 (Ref)	1 (Ref)	1 (Ref)
	0–12	RVX	6	256	23.46 (8.61–51.06)	0.48 (0.21–1.08)	0.62 (0.26–1.49)	0.59 (0.26–1.32)	0.68 (0.20–2.33)
		LMWH	181	3682	49.16 (42.26–56.86)	1 (Ref)	1 (Ref)	1 (Ref)	1 (Ref)
	0–24	RVX	10	460	21.73 (10.42–39.97)	0.62 (0.33–1.17)	0.71 (0.35–1.46)	0.81 (0.43–1.52)	0.81 (0.28–2.32)
		LMWH	218	6063	35.96 (31.34–41.06)	1 (Ref)	1 (Ref)	1 (Ref)	1 (Ref)
	Overall	RVX	17	872	19.51 (11.36–31.23)	0.80 (0.50–1.30)	1.04 (0.60–1.82)	1.13 (0.70–1.84)	1.27 (0.53–3.02)
		LMWH	273	10,918	25.00 (22.13–28.15)	1 (Ref)	1 (Ref)	1 (Ref)	1 (Ref)
All-cause mortality	0–3	RVX	13	68	190.4 (101.4–325.6)	0.21 (0.12–0.36)	0.47 (0.27–0.83)	Competing risk	
		LMWH	955	1161	822.7 (771.3–876.5)	1 (Ref)	1 (Ref)		
	0–6	RVX	21	134	156.7 (97.01–239.6)	0.20 (0.13–0.31)	0.41 (0.26–0.65)		
		LMWH	1522	2127	715.4 (679.9–752.3)	1 (Ref)	1 (Ref)		
	0–12	RVX	38	259	146.8 (103.9–201.5)	0.24 (0.18–0.33)	0.48 (0.34–0.67)		
		LMWH	2125	3757	565.6 (541.8–590.2)	1 (Ref)	1 (Ref)		
	0–24	RVX	54	468	115.3 (86.59–150.4)	0.26 (0.20–0.34)	0.48 (0.36–0.64)		
		LMWH	2628	6220	422.5 (406.5–439.0)	1 (Ref)	1 (Ref)		
	Overall	RVX	70	896	78.11 (60.89–98.68)	0.28 (0.22–0.35)	0.50 (0.39–0.64)		
		LMWH	3084	11,309	272.7 (263.2–282.5)	1 (Ref)	1 (Ref)		

CI, confidence interval; HR, hazard ratio; IR, incidence rate; LMWH, low molecular weight heparin; PY, person-years; RVX, rivaroxaban; VTE, venous thromboembolism; Ref. Reference category. ^a^Adjusted for sex and age

For rVTE comparing rivaroxaban with LMWH, the weighted HR was similar for different follow-up times and no results were statistically significant. For rivaroxaban, 12 out of 25 cases of rVTE happened during the 3-month follow-up, compared with 230 out of 473 for LMWH. When considering death as a competing risk, the sub-HRs were similar to the corresponding HRs, but with broader CIs. Both HR and sub-HR of major bleeding favoured rivaroxaban, and no results were statistically significant. For the comparison of mortality between rivaroxaban and LMWH all results were statistically significant, favouring rivaroxaban with point estimates close to 0.5, which did not change much for different follow-up times.

The on-treatment analysis showed consistent results for the three outcomes, as shown in Supplementary Table S4.

## Discussion

The risk of rVTE appears to be similar in patients treated with rivaroxaban compared with LMWH. The results for major bleeding appeared similar to those of rVTE. Sub-HRs considering death as competing events were similar to the HRs from Cox regression. A consistently lower all-cause mortality for rivaroxaban compared with LMWH was observed for all follow-up times.

In observational studies, there are discrepancies in the HRs comparing rivaroxaban with LMWH regarding rVTE, major bleeding, and mortality. Costa et al. used the US Surveillance, Epidemiology, and End Results–Medicare-linked data, including patients with CAT who were admitted to hospital or treated in an emergency department and subsequently prescribed rivaroxaban or LMWH for outpatient anticoagulation. Costa et al. applied a PS-matched approach. No differences were observed for major bleeding with a HR 1.01 (95% CI 0.50–2.01) and a mortality HR 0.87 (95% CI 0.70–1.07), but rivaroxaban reduced rVTE with a HR 0.37 (95% CI 0.15–0.95) [27]. An observational study by Coleman et al., showed a HR for rVTE of 0.69 (95% CI 0.51–0.92), without differences in major bleeding (HR 0.79 [95% CI 0.55–1.13]) and all-cause mortality (HR 1.07 [95% CI 0.85–1.35]) for rivaroxaban compared to LMWH [18]. In agreement, another observational study by Streiff et al. included 707 patients with CAT treated with rivaroxaban and 660 patients treated with LMWH for 3 months using data from claims, showing that rVTE was significantly lower for rivaroxaban with a HR 0.72 (95% CI 0.52–0.95), and with similar rates of major bleeding [15].

A retrospective cohort study of 4000 individuals with CAT comparing DOACs with LMWH by Riaz et al., additionally reported a higher risk of rVTE, higher risk of major bleeding, and also an increased risk of all-cause mortality (HR, 1.61; 95% CI, 1.15–2.25) with LMWH [28]. A meta-analysis of real-world data and randomized controlled trials (RCTs) comparing rivaroxaban with LMWH in patients with CAT by Mohamed et al., showed fewer rVTE events, lower all-cause mortality, similar major bleeding risk, and a higher risk of clinically relevant non-major bleeding events for rivaroxaban [16]. Another meta-analysis by Song et al., found similar results considering rVTE and bleeding after 12 months of follow-up [17]. For comparison, the weighted adjusted HRs in the current study were 0.91 for rVTE and 0.65 for major bleeding, both closer to 1 and not statistically significant, and with a HR of 0.57 for all-cause mortality being significant. The reported differences among studies may be related to the design, the included population, the definition of exposure, and/or differences in outcomes between RCTs and observational studies.

In the current study, upper gastrointestinal malignancies were excluded due to higher bleeding risk. Given that individual decisions on anticoagulation treatment are done considering bleeding and thrombosis risk for each patient, this potential restriction to external validity excluding patients with higher risk of bleeding may be consistent with clinical practice in a real-world setting. Different cancer subpopulations also showed some discrepancies. Rivaroxaban compared with LMWH had similar rVTE risk, but higher risk of bleeding in patients with CAT and active locally advanced unresectable or metastatic cancers, especially upper gastrointestinal tract and hepatopancreatobiliary cancers [29]. These findings were also supported by a meta-analysis of gastrointestinal cancers [30]. No difference was observed in rVTE, major bleeding, or all-cause mortality in long-term treatment with rivaroxaban compared with dalteparin in patients with CAT associated with lung [31] or gynaecologic cancer [32].

The lower all-cause mortality with rivaroxaban compared with LMWH in the current study is consistent with some published observational studies [13, 14], but inconsistent with results from RCTs [12, 33, 34].This discrepancy suggests that despite adjusting for confounders, there are unmeasured confounders such as systemic treatment for cancer, severity of cancer and other comorbidities, lifestyle factors, family history of VTE, indications for prescriptions and physicians’ choice of treatment. Hence, interpretation of results, specifically regarding mortality, should be made with caution.

Effectiveness and safety outcomes in the current study are supportive of current treatment guidelines for VTE in patients with cancer that recommend the use of DOACs or LMWH for initial treatment within the first week and for short-term treatment (3 to 6 months) [8, 9]. For longer-term treatment, persistence is likely to be higher with DOACs than LMWH due to easier administration and proven higher treatment adherence [11, 35], but there is a paucity of evidence comparing the efficacy and safety of DOACs beyond that first 6–12 months of therapy with LMWH. Treatment decisions should balance benefits and harms, integrating individual values, preferences, and available alternative strategies [11].

Strengths of the current study include long-term follow-up with no loss to follow-up, with relatively large sample size. We included all CAT cases in Sweden, without any selection, allowing estimation of an overall risk of rVTE and bleeding in patients with cancers not associated with high bleeding risk consistent with the ISTH’s recommendation. CAT populations have increased mortality, and hence death was handled as a competing event for rVTE and bleeding in additional analyses.

Some limitations include the following: first, exposure to drugs provided during hospitalizations were not captured in this study, unless they were dispensed to the patient through their personal identifier. Second, the relatively small sample size, particularly in the rivaroxaban group, influenced the precision of the HRs and the sub-HRs resulting in broad CIs making small effect sizes hard to interpret. Third, it is possible that residual confounding from unmeasured confounders, for example, comorbidities and cancer prognosis, are the main driver in the observed difference in mortality between the exposure groups. Fourth, channelling bias may still have an effect on the results [36]. As an example, if physicians preferentially prescribe LMWH over DOACs to patients with a high bleeding risk, the resulting relative risk estimate comparing DOACs with LMWH could be biased in direction towards the null. Despite this, the comparison groups were balanced by PS overlap weighting, including all available identified confounders. Fifth, the time period covered by this study may not reflect current cancer therapies or how DOACs are currently used, since the pivotal RCTs comparing DOACs with LMWH were published late (mid-2018) in the study period. Therefore, it is possible that rivaroxaban was used in selected cancer patients.

## Conclusion

In patients with CAT who do not have a cancer with a high risk of bleeding, treatment with rivaroxaban appears to perform similarly to LMWH for rVTE and major bleeding at 3, 6, 12, and 24 months of follow-up. Rivaroxaban was associated with a lower risk of death as compared with LMWH. However, results for mortality should be interpreted with caution since this observation may be a result of residual confounding.

### Electronic supplementary material

Below is the link to the electronic supplementary material.

Supplementary Material 1
