# Supplementary material for: Comparison of rivaroxaban and low molecular weight heparin in the treatment of cancer-associated venous thromboembolism: a Swedish national population-based register study
Source: J Thromb Thrombolysis. 2024 May 12;57(6):973–83. doi: 10.1007/s11239-024-02992-1 (PMC11315776; doi:10.1007/s11239-024-02992-1)
Supplement: Supplementary file 1 — Supplementary Material 1 [file 11239_2024_2992_MOESM1_ESM.docx]

**SUPPLEMENTARY INFORMATION**

**Comparison of rivaroxaban and low molecular weight heparin in the treatment of cancer-associated venous thromboembolism: a Swedish national population-based register study**

Marie Linder^1^; Anders Ekbom^1^; Kai Vogtländer^2^; Yanina Balabanova^2^; Cecilia Becattini^3^; Marc Carrier^4^; Alexander T. Cohen^5^; Craig I. Coleman^6^; Alok A. Khorana^7^; Agnes Y.Y. Lee^8^; George Psaroudakis^2^; Khaled Abdelgawwad^2^; Marcela Rivera^9^; Bernhard Schaefer^2^; Gunnar Brobert^10^ ;Diego Hernan Giunta^1^

^1^Department of Medicine Solna, Centre for Pharmacoepidemiology, Karolinska Institutet, Karolinska University Hospital, Stockholm, Sweden

^2^Bayer AG, Berlin, Germany

^3^Department of Internal and Emergency Medicine – Stroke Unit, University of Perugia, Perugia, Italy

^4^Department of Medicine, Ottawa Hospital Research Institute at the University of Ottawa, Ottawa, Canada

^5^Department of Haematological Medicine, Guy’s and St Thomas’ NHS Foundation Trust, King’s College London, London, United Kingdom

^6^School of Pharmacy, University of Connecticut, Storrs, Connecticut, USA

^7^Cleveland Clinic and Case Comprehensive Cancer Center, Cleveland, Ohio, USA

^8^University of British Columbia and BC Cancer, Vancouver, Canada

^9^Bayer AG, Berlin, Germany at the time of study conduct, currently affiliated to Janssen Research and Development, Barcelona, Spain

^10^Consultant for Bayer AG, Berlin, Germany

**Contact information for corresponding author:**

Name: Marie Linder

Email: marie.linder@ki.se

**Journal:** *Journal of Thrombosis and Thrombolysis*

Supplementary tables

**Supplementary Table S1** Definitions and codes for excluded malignancies, exposure and outcomes

|  | **ICD-10, NSCP codes** | **ATC codes** |
| --- | --- | --- |
| **Exclusion criteria** |  |  |
| **Excluded malignancies** |  |  |
| CANCER LIP | C00 |  |
| CANCER UPPER GI | C15–C16 |  |
| CANCER NMSC | C44 |  |
| CANCER IMMUNO | C88 |  |
| CANCER LEUKEMIA | C91-C95 |  |
| NON-RESECTIONAL PROCEDURES [1] | JGA32, JGA35, JGA52, JGA58, JGA73, JGA75, JGA76, JGA96, JGA97, JGA98, JGW, JFA68, JFA83, JFA84, JFA96, JFA97, JFC, JFF10, JFF11, JFF13, JFF20, JFF21, JFF23, JFF24, JFF26, JFF27, JFF30, JFF31, JFW |  |
| **Other exclusion criteria** |  |  |
| ATRIAL FIBRILLATION | I48 |  |
| TOTAL HIP REPLACEMENT | NFB0, NFB1, NFB20, NFB30, NFB40, NFB59, NFB99, NFC0, NFC1, NFC2, NFC3, NFC4, NFC59, NFC99 |  |
| TOTAL KNEE REPLACEMENT | NGB0, NGB1, NGB20, NGB30, NGB40, NGB59, NGB99, NGC0, NGC1, NGC2, NGC3, NGC4, NGC59, NGC99 |  |
| ACUTE CORONARY SYNDROME | I24 |  |
| **Exposure** |  |  |
| DOACs  Rivaroxaban  Dabigatran  Apixaban  Edoxaban |  | B01AF01  B01AE07  B01AF02  B01AF03 |
| LMWH  Enoxaparin  Dalteparin  Nadroparin  Tinzaparin |  | B01AB05  B01AB04  B01AB06  B01AB10 |
| **Outcomes** |  |  |
| Recurrent VTE | I26#, I801, I802, I803, I828, I829, I843 |  |
| Major bleeding | A985, D62#, D683, G951, I230, I312, I600, I601, I602, I603, I604, I605, I606, I607, I608, I609, I610, I611, I612, I613, I614, I615, I616, I618, I619, I620, I621, I629, I850, J942, J958, K22#, K25#, K26#, K27#, K28#, K29#, K57#, K92#, N421, N857, N920, N923, N926, N930, N938, N939, N998, R04#, R58# |  |

**Supplementary Table S2** Baseline characteristics, frequency (percentage), before and after PS weighting

| **Variable** | **Value** | **Rivaroxaban** | **LMWH** | **Rivaroxaban** | **LMWH** |
| --- | --- | --- | --- | --- | --- |
| Total number |  | 283 | 5181 | 200 | 200 |
| Age | <65 | 78 (28%) | 1739 (34%) | 57 (28%) | 57 (28%) |
|  | >=65 | 205 (72%) | 3442 (66%) | 143 (72%) | 143 (72%) |
| Sex | Female | 137 (48%) | 2952 (57%) | 103 (51%) | 103 (51%) |
|  | Male | 146 (52%) | 2229 (43%) | 97 (49%) | 97 (49%) |
| Inclusion year | 2013 | 12 (4%) | 766 (15%) | 7 (3%) | 7 (3%) |
|  | 2014 | 40 (14%) | 744 (14%) | 22 (11%) | 22 (11%) |
|  | 2015 | 49 (17%) | 746 (14%) | 36 (18%) | 36 (19%) |
|  | 2016 | 37 (13%) | 753 (15%) | 32 (16%) | 32 (16%) |
|  | 2017 | 56 (20%) | 746 (14%) | 33 (17%) | 33 (17%) |
|  | 2018 | 50 (18%) | 769 (15%) | 36 (18%) | 36 (18%) |
|  | 2019 | 39 (14%) | 657 (13%) | 34 (17%) | 34 (17%) |
| Type of VTE | DVT | 102 (36%) | 2122 (41%) | 73 (36%) | 73 (36%) |
|  | PE | 171 (60%) | 2828 (55%) | 120 (60%) | 120 (60%) |
|  | Both | 10 (4%) | 231 (4%) | 7 (3%) | 7 (3%) |
| Cancer type | Oral cavity and pharynx | 2 (0.7%) | 67 (1%) | 2 (1%) | 2 (1%) |
|  | Digestive organs | 52 (18%) | 1561 (30%) | 42 (21%) | 42 (21%) |
|  | Respiratory and intrathoracic organs | 14 (5%) | 946 (18%) | 12 (6%) | 12 (6%) |
|  | Bone and articular cartilage | 0 (0.00%) | 11 (0.2%) | 0 (0.00%) | 0 (0.00%) |
|  | Malignant melanoma | 37 (13%) | 51 (1%) | 15 (8%) | 15 (8%) |
|  | Mesothelial and soft tissue | 2 (0.7%) | 57 (1%) | 2 (0.9%) | 2 (0.9%) |
|  | Breast | 30 (11%) | 606 (12%) | 24 (12%) | 24 (12%) |
|  | Female genital organs | 14 (5%) | 382 (7%) | 12 (6%) | 12 (6%) |
|  | Male genital organs | 58 (20%) | 265 (5%) | 33 (17%) | 33 (17%) |
|  | Urinary tract | 17 (6%) | 321 (6%) | 14 (7%) | 14 (7%) |
|  | Eye, brain, and other parts of central | 27 (10%) | 350 (7%) | 22 (11%) | 22 (11%) |
|  | Thyroid and other endocrine glands | 11 (4%) | 30 (0.6%) | 6 (3%) | 6 (3%) |
|  | Ill-defined, secondary and unspecified | 1 (0.4%) | 188 (4%) | 1 (0.5%) | 1 (0.5%) |
|  | Lymphoid, haematopoietic and related tissue | 18 (6%) | 346 (7%) | 15 (7%) | 15 (7%) |
| Aggregated TNM | 0 | 30 (11%) | 159 (3%) | 17 (9%) | 17 (9%) |
|  | 1 | 70 (25%) | 718 (14%) | 44 (22%) | 44 (22%) |
|  | 2 | 58 (20%) | 820 (16%) | 38 (19%) | 38 (19%) |
|  | 3 | 26 (9%) | 1080 (21%) | 22 (11%) | 22 (11%) |
|  | 4 | 20 (7%) | 914 (18%) | 17 (9%) | 17 (9%) |
|  | Missing | 79 (28%) | 1490 (29%) | 62 (31%) | 62 (31%) |
| Hospital duration before index date | 0 weeks | 158 (56%) | 2443 (47%) | 103 (52%) | 103 (52%) |
|  | 1–2 weeks | 84 (30%) | 1779 (34%) | 65 (33%) | 65 (33%) |
|  | 3–10 weeks | 38 (13%) | 904 (17%) | 31 (16%) | 31 (16%) |
|  | >10 weeks | 3 (1%) | 55 (1%) | 0 (0.2%) | 0 (0.2%) |
| ACE inhibitor | Yes | 64 (23%) | 895 (17%) | 40 (20%) | 40 (20%) |
| Angiotensin blockers | Yes | 60 (21%) | 973 (19%) | 39 (20%) | 39 (20%) |
| Antidepressants | Yes | 26 (9%) | 345 (7%) | 18 (9%) | 18 (9%) |
| Antidiabetics | Yes | 39 (14%) | 677 (13%) | 29 (14%) | 29 (14%) |
| Antiplatelet | Yes | 142 (50%) | 2230 (43%) | 97 (48%) | 97 (48%) |
| Antivirals | Yes | 8 (3%) | 234 (5%) | 6 (3%) | 6 (3%) |
| Benzodiazepines | Yes | 52 (18%) | 1012 (20%) | 41 (20%) | 41 (20%) |
| Beta blockers | Yes | 83 (29%) | 1342 (26%) | 58 (29%) | 58 (29%) |
| Calcium channel blockers | Yes | 64 (23%) | 1064 (21%) | 46 (23%) | 46 (23%) |
| Cancer treatment | Yes | 34 (12%) | 617 (12%) | 28 (14%) | 28 (14%) |
| Contraceptive/oestrogen | Yes | 3 (1%) | 67 (1%) | 3 (1%) | 3 (1%) |
| Corticosteroids | Yes | 97 (34%) | 2612 (50%) | 75 (38%) | 75 (38%) |
| Digitoxin | Yes | 1 (0.4%) | 36 (0.7%) | 1 (0.5%) | 1 (0.5%) |
| H_2_-receptor antagonist | Yes | 3 (1%) | 107 (2%) | 3 (1%) | 3 (1%) |
| Hormone replacement therapy | Yes | 22 (8%) | 460 (9%) | 17 (8%) | 17 (8%) |
| Loop diuretics | Yes | 42 (15%) | 604 (12%) | 29 (14%) | 29 (14%) |
| Macrolides | Yes | 1 (0.4%) | 70 (1%) | 1 (0.3%) | 1 (0.3%) |
| Moderate CYP3A4 inducers | Yes | 0 (0.00%) | 62 (1%) | 0 (0.00%) | 0 (0.00%) |
| Moderate CYP3A4 inhibitors | Yes | 20 (7%) | 629 (12%) | 16 (8%) | 16 (8%) |
| NSAIDs | Yes | 58 (20%) | 1287 (25%) | 44 (22%) | 44 (22%) |
| SNRI | Yes | 8 (3%) | 156 (3%) | 5 (3%) | 5 (3%) |
| SSRI | Yes | 30 (11%) | 500 (10%) | 21 (11%) | 21 (11%) |
| Statins | Yes | 75 (27%) | 1246 (24%) | 49 (24%) | 49 (24%) |
| Strong CYP3A4 inhibitors | Yes | 0 (0.00%) | 27 (0.5%) | 0 (0.00%) | 0 (0.00%) |
| Testosterone | Yes | 19 (7%) | 180 (3%) | 12 (6%) | 12 (6%) |
| Thiazides | Yes | 18 (6%) | 315 (6%) | 14 (7%) | 14 (7%) |
| Acute bronchitis | Yes | 2 (0.7%) | 86 (2%) | 2 (0.9%) | 2 (0.9%) |
| Anaemia | Yes | 28 (10%) | 863 (17%) | 23 (12%) | 23 (12%) |
| Angina pectoris | Yes | 21 (7%) | 335 (6%) | 14 (7%) | 14 (7%) |
| Asthma | Yes | 19 (7%) | 301 (6%) | 13 (7%) | 13 (7%) |
| Bronchiectasis | Yes | 0 (0.00%) | 11 (0.2%) | 0 (0.00%) | 0 (0.00%) |
| Cellulitis/skin infection | Yes | 18 (6%) | 287 (6%) | 13 (6%) | 13 (6%) |
| Cerebrovascular disease | Yes | 31 (11%) | 469 (9%) | 20 (10%) | 20 (10%) |
| Coronary heart failure | Yes | 25 (9%) | 268 (5%) | 15 (8%) | 15 (8%) |
| Chronic inflammatory disease | Yes | 12 (4%) | 217 (4%) | 8 (4%) | 8 (4%) |
| Coagulation disorder | Yes | 10 (4%) | 150 (3%) | 7 (4%) | 7 (4%) |
| Dementia | Yes | 5 (2%) | 50 (1%) | 3 (1%) | 3 (1%) |
| Depression | Yes | 14 (5%) | 308 (6%) | 10 (5%) | 10 (5%) |
| Diabetes | Yes | 42 (15%) | 717 (14%) | 31 (15%) | 31 (15%) |
| Diverticulitis | Yes | 24 (8%) | 387 (7%) | 16 (8%) | 16 (8%) |
| Gastric bleeding | Yes | 13 (5%) | 359 (7%) | 10 (5%) | 10 (5%) |
| Gastritis | Yes | 13 (5%) | 273 (5%) | 8 (4%) | 8 (4%) |
| GERD | Yes | 17 (6%) | 302 (6%) | 11 (6%) | 11 (6%) |
| Heart failure | Yes | 25 (9%) | 268 (5%) | 15 (8%) | 15 (8%) |
| Hiatus hernia | Yes | 14 (5%) | 315 (6%) | 10 (5%) | 10 (5%) |
| Hypertension | Yes | 136 (48%) | 2150 (41%) | 96 (48%) | 96 (48%) |
| Hyperlipidaemia | Yes | 32 (11%) | 624 (12%) | 22 (11%) | 22 (11%) |
| Hypothyroidism | Yes | 14 (5%) | 270 (5%) | 12 (6%) | 12 (6%) |
| IBD | Yes | 6 (2%) | 100 (2%) | 4 (2%) | 4 (2%) |
| Influenza | Yes | 3 (1%) | 20 (0.4%) | 2 (0.8%) | 2 (0.8%) |
| Internal bleeding | Yes | 4 (1%) | 70 (1%) | 2 (0.8%) | 2 (0.8%) |
| Ischaemic heart disease | Yes | 24 (8%) | 422 (8%) | 17 (8%) | 17 (8%) |
| Kidney disease | Yes | 4 (1%) | 55 (1%) | 2 (1%) | 2 (1%) |
| Liver disease | Yes | 1 (0.4%) | 84 (2%) | 1 (0.3%) | 1 (0.3%) |
| Myocardial infarction | Yes | 20 (7%) | 326 (6%) | 14 (7%) | 14 (7%) |
| Osteomyelitis | Yes | 3 (1%) | 12 (0.2%) | 1 (0.7%) | 1 (0.7%) |
| Osteoporosis | Yes | 15 (5%) | 173 (3%) | 9 (5%) | 9 (5%) |
| Other bleeding | Yes | 3 (1%) | 53 (1%) | 3 (1%) | 3 (1%) |
| Peripheral arterial disease | Yes | 14 (5%) | 190 (4%) | 10 (5%) | 10 (5%) |
| Rheumatoid arthritis | Yes | 21 (7%) | 353 (7%) | 17 (8%) | 17 (8%) |
| Sleep apnoea | Yes | 17 (6%) | 239 (5%) | 12 (6%) | 12 (6%) |
| Systemic embolism | Yes | 6 (2%) | 56 (1%) | 4 (2%) | 4 (2%) |
| Thrombosis | Yes | 5 (2%) | 38 (0.7%) | 4 (2%) | 4 (2%) |
| Total bleeding | Yes | 29 (10%) | 591 (11%) | 20 (10%) | 20 (10%) |
| Thrombophilia | Yes | 5 (2%) | 38 (0.7%) | 4 (2%) | 4 (2%) |
| Upper UTI | Yes | 26 (9%) | 333 (6%) | 19 (9%) | 19 (9%) |
| Urogenital bleeding | Yes | 9 (3%) | 146 (3%) | 6 (3%) | 6 (3%) |
| Venous insufficiency | Yes | 4 (1%) | 32 (0.6%) | 2 (1%) | 2 (1%) |
| Coronary procedures | Yes | 15 (5%) | 265 (5%) | 10 (5%) | 10 (5%) |
| Central venous catheter | Yes | 7 (2%) | 351 (7%) | 7 (3%) | 7 (3%) |
| Major surgery | Yes | 247 (87%) | 4226 (82%) | 174 (87%) | 174 (87%) |
| Alcohol proxy | Yes | 5 (2%) | 138 (3%) | 4 (2%) | 4 (2%) |
| Smoking proxy | Yes | 23 (8%) | 625 (12%) | 17 (9%) | 17 (9%) |
| Education | Elementary school | 97 (34%) | 1484 (29%) | 71 (36%) | 71 (36%) |
|  | Highschool | 114 (40%) | 2307 (45%) | 80 (40%) | 80 (40%) |
|  | College/university | 63 (22%) | 1282 (25%) | 43 (22%) | 43 (22%) |
|  | Postgraduate | 8 (3%) | 55 (1%) | 6 (3%) | 6 (3%) |
|  | Missing | 1 (0.4%) | 53 (1%) | 0 (0.00%) | 0 (0.00%) |
| Employment | Employed | 87 (31%) | 1745 (34%) | 64 (32%) | 64 (32%) |
|  | Not employed | 196 (69%) | 3432 (66%) | 136 (68%) | 136 (68%) |
|  | Missing | 0 (0.00%) | 4 (0.08%) | 0 (0.00%) | 0 (0.00%) |
| Income quintiles | Low | 61 (22%) | 1109 (21%) | 44 (22%) | 44 (22%) |
|  | Low–mid | 58 (20%) | 1055 (20%) | 43 (21%) | 43 (21%) |
|  | Mid | 59 (21%) | 1080 (21%) | 40 (20%) | 40 (20%) |
|  | Mid–high | 52 (18%) | 985 (19%) | 38 (19%) | 38 (19%) |
|  | High | 53 (19%) | 948 (18%) | 36 (18%) | 36 (18%) |
|  | Missing | 0 (0.00%) | 4 (0.08%) | 0 (0.00%) | 0 (0.00%) |
| Marital status | Married | 145 (51%) | 2754 (53%) | 100 (50%) | 100 (50%) |
|  | Divorced | 43 (15%) | 914 (18%) | 31 (16%) | 31 (16%) |
|  | Unmarried | 45 (16%) | 867 (17%) | 34 (17%) | 34 (17%) |
|  | Widowed | 50 (18%) | 642 (12%) | 35 (17%) | 35 (17%) |
|  | Missing | 0 (0.00%) | 4 (0.08%) | 0 (0.00%) | 0 (0.00%) |
| Region | Predominantly urban | 12 (4%) | 1002 (19%) | 10 (5%) | 11 (5%) |
|  | Intermediate | 122 (43%) | 1544 (30%) | 85 (42%) | 84 (42%) |
|  | Predominantly rural | 149 (53%) | 2631 (51%) | 105 (52%) | 104 (52%) |
|  | Missing | 0 (0.00%) | 4 (0.08%) | 0 (0.00%) | 0 (0.00%) |

ACE, angiotensin-converting enzyme; DVT, deep vein thrombosis; IBD, inflammatory bowel disease; LMWH, low molecular weight heparin; NSAID, non-steroidal anti-inflammatory drug; PE, pulmonary embolism; SNRI, serotonin and norepinephrine reuptake inhibitors; SSRI, selective serotonin reuptake inhibitor; UTI, urinary tract infection; VTE, venous thromboembolism.

**Supplementary Table S3** Cancer groups

| Concept | ICD-code | Content | Grouping/Naming |
| --- | --- | --- | --- |
| ORAL | C01-C14 | Malignant neoplasm of base of tongue  Malignant neoplasm of other and unspecified parts of tongue  Malignant neoplasm of gum  Malignant neoplasm of floor of mouth  Malignant neoplasm of palate  Malignant neoplasm of other and unspecified parts of mouth  Malignant neoplasm of parotid gland  Malignant neoplasm of other and unspecified major salivary glands  Malignant neoplasm of tonsil  Malignant neoplasm of oropharynx  Malignant neoplasm of nasopharynx  Malignant neoplasm of piriform sinus  Malignant neoplasm of hypopharynx  Malignant neoplasm of other and ill-defined sites in the lip, oral cavity and pharynx | 01. oral cavity and pharynx |
| SMALL INTENSTINE | C17 | Malignant neoplasm of small intestine | 02. digestive organs |
| COLORECTAL | C18-C21 | Malignant neoplasm of colon  Malignant neoplasm of rectosigmoid junction  Malignant neoplasm of rectum  Malignant neoplasm of anus and anal canal | 02. digestive organs |
| LIVER | C22-C24 | Malignant neoplasm of liver and intrahepatic bile ducts  Malignant neoplasm of gallbladder  Malignant neoplasm of other and unspecified parts of biliary tract | 02. digestive organs |
| PANCREATIC | C25 | Malignant neoplasm of pancreas | 02. digestive organs |
| NASAL LARYNX | C30-C33 | Malignant neoplasm of nasal cavity and middle ear  Malignant neoplasm of accessory sinuses  Malignant neoplasm of larynx  Malignant neoplasm of trachea | 03. respiratory and intrathoracic organs |
| LUNG | C34 | Malignant neoplasm of bronchus and lung | 03. respiratory and intrathoracic organs |
| THYMUS | C37 | Malignant neoplasm of thymus | 03. respiratory and intrathoracic organs |
| HEART PLEURA | C38 | Malignant neoplasm of heart, mediastinum and pleura | 03. respiratory and intrathoracic organs |
| BONE | C40-C41 | Malignant neoplasm of bone and articular cartilage of limbs  Malignant neoplasm of bone and articular cartilage of other and unspecified sites | 04. bone and articular cartilage |
| MELANOMA | C43 | Malignant melanoma of skin | 05. Malignant melanoma |
| SOFT TISSUE | C45-C49 | Mesothelioma  Kaposi sarcoma  Malignant neoplasm of peripheral nerves and autonomic nervous system  Malignant neoplasm of retroperitoneum and peritoneum  Malignant neoplasm of other connective and soft tissue | 06. mesothelial and soft tissue |
| BREAST | C50 | Malignant neoplasm of breast | 07. Breast |
| GYN | C51-C58 | Malignant neoplasm of vulva  Malignant neoplasm of vagina  Malignant neoplasm of cervix uteri  Malignant neoplasm of corpus uteri  Malignant neoplasm of uterus, part unspecified  Malignant neoplasm of ovary  Malignant neoplasm of other and unspecified female genital organs  Malignant neoplasm of placenta | 08. female genital organs |
| MALE GENITAL | C60-C63 | Malignant neoplasm of penis  Malignant neoplasm of testis  Malignant neoplasm of other and unspecified male genital organs | 09. male genital organs |
| PROSTATE | C61 | Malignant neoplasm of prostate | 09. male genital organs |
| KIDNEY | C64 | Malignant neoplasm of kidney, except renal pelvis | 10. urinary tract |
| URINARY | C65, C66, C68 | Malignant neoplasm of renal pelvis  Malignant neoplasm of ureter  Malignant neoplasm of other and unspecified urinary organs | 10. urinary tract |
| BLADDER | C67 | Malignant neoplasm of bladder | 10. urinary tract |
| EYE | C69 | Malignant neoplasm of eye and adnexa | 11. eye, brain and other parts of central nervous system |
| CNS | C70-C72 | Malignant neoplasm of meninges  Malignant neoplasm of brain  Malignant neoplasm of spinal cord, cranial nerves and other parts of central nervous system | 11. eye, brain and other parts of central nervous system |
| ENDOCRINE | C73-C75 | Malignant neoplasm of thyroid gland  Malignant neoplasm of adrenal gland  Malignant neoplasm of other endocrine glands and related structures | 12. thyroid and other endocrine glands |
| OTHER | C26, C39, C76, C80, C96 | Malignant neoplasm of other and ill-defined digestive organs  Malignant neoplasm of other and ill-defined sites in the respiratory system and intrathoracic organs  Malignant neoplasm of other and ill-defined sites  Malignant neoplasm, without specification of site  Other and unspecified malignant neoplasms of lymphoid, haematopoietic and related tissue | 13. ill-defined, secondary and unspecified sites |
| LYMPH NODES | C77 | Secondary and unspecified malignant neoplasm of lymph nodes | 13. ill-defined, secondary and unspecified sites |
| METASTATIC | C78-C79 | Secondary malignant neoplasm of respiratory and digestive organs  Secondary malignant neoplasm of other and unspecified sites | 13. ill-defined, secondary and unspecified sites |
| LYMPHOMA | C81-C85 | Hodgkin lymphoma  Follicular lymphoma  Non-follicular lymphoma  Mature T/NK-cell lymphomas  Other and unspecified types of non-Hodgkin lymphoma | 14. lymphoid, hematopoietic and related tissue |
| MYELOM | C90 | Multiple myeloma and malignant plasma cell neoplasms | 14. lymphoid, hematopoietic and related tissue; |

**Supplementary Table S4** Crude and adjusted HRs from Cox regression and subhazards from Fine-Gray regression for recurrent VTE and major bleeding; rivaroxaban vs LMWH; OT exposure definition

| **Outcome** | **Months follow-up** | **Group** | **Total**  **(N)** | **Events**  **(N)** | **Person-years** | **IR (CI) per 1000 PY** | **Unweighted**  **HR* (CI)** | **Weighted**  **sub-HR (CI)** | **Unweighted**  **sub-HR^a^ (CI)** | **Weighted**  **sub-HR (CI)** |
| --- | --- | --- | --- | --- | --- | --- | --- | --- | --- | --- |
| Recurrent VTE | 0–3 | RVX | 283 | 11 | 59.35 | 185.3  (92.52–331.6) | 0.87  (0.48–1.61) | 0.80  (0.40–1.63) | 0.90  (0.49–1.66) | 0.82  (0.33–2.05) |
|  |  | LMWH | 5181 | 225 | 1046.95 | 214.9  (187.7–244.9) | 1.00 (REF) | 1.00 (REF) | 1.00 (REF) | 1.00 (REF) |
|  | 0–6 | RVX | 283 | 11 | 104.96 | 104.8  (52.31–187.5) | 0.76  (0.41–1.40) | 0.70  (0.35–1.42) | 0.81 (0.44–1.48) | 0.72  (0.30–1.77) |
|  |  | LMWH | 5181 | 253 | 1753.13 | 144.3  (127.1–163.2) | 1.00 (REF) | 1.00 (REF) | 1.00 (REF) | 1.00 (REF) |
|  | 0–12 | RVX | 283 | 11 | 149.65 | 73.51  (36.69–131.5) | 0.65  (0.35–1.20) | 0.63  (0.32–1.25) | 0.72  (0.39–1.32) | 0.66  (0.27–1.57) |
|  |  | LMWH | 5181 | 291 | 2509.54 | 116.0  (103.0–130.1) | 1.00 (REF) | 1.00 (REF) | 1.00 (REF) | 1.00 (REF) |
|  | 0–24 | RVX | 283 | 11 | 175.57 | 62.65  (31.28–112.1) | 0.61  (0.33–1.13) | 0.61  (0.31–1.21) | 0.70  (0.38–1.28) | 0.64  (0.27–1.51) |
|  |  | LMWH | 5181 | 308 | 2893.88 | 106.4  (94.88–119.0) | 1.00 (REF) | 1.00 (REF) | 1.00 (REF) | 1.00 (REF) |
|  | Overall | RVX | 283 | 11 | 180.59 | 60.91  (30.41–109.0) | 0.61  (0.33–1.13) | 0.61  (0.31–1.21) | 0.70  (0.38–1.28) | 0.64  (0.27–1.51) |
|  |  | LMWH | 5181 | 310 | 3061.88 | 101.2  (90.29–113.2) | 1.00 (REF) | 1.00 (REF) | 1.00 (REF) | 1.00 (REF) |
| Major bleeding | 0–3 | RVX | 283 | 1 | 61.13 | 16.36  (0.41–91.15) | 0.19  (0.03–1.37) | 0.18  (0.02–1.39) | 0.21  (0.03–1.48) | 0.19  (0.01–3.26) |
|  |  | LMWH | 5181 | 88 | 1073.82 | 81.95  (65.73–101.0) | 1.00 (Ref) | 1.00 (Ref) | 1.00 (Ref) | 1.00 (Ref) |
|  | 0–6 | RVX | 283 | 2 | 108.52 | 18.43  (2.23–66.57) | 0.24  (0.06–0.97) | 0.28  (0.07–1.20) | 0.28  (0.07–1.12) | 0.30  (0.04–2.10) |
|  |  | LMWH | 5181 | 131 | 1798.26 | 72.85  (60.91–86.44) | 1.00 (Ref) | 1.00 (Ref) | 1.00 (Ref) | 1.00 (Ref) |
|  | 0–12 | RVX | 283 | 3 | 154.48 | 19.42  (4.00–56.75) | 0.31  (0.10–0.96) | 0.32  (0.09–1.09) | 0.37  (0.12–1.15) | 0.35  (0.06–2.00) |
|  |  | LMWH | 5181 | 156 | 2576.28 | 60.55  (51.42–70.84) | 1.00 (Ref) | 1.00 (Ref) | 1.00 (Ref) | 1.00 (Ref) |
|  | 0–24 | RVX | 283 | 5 | 179.67 | 27.83  (9.04–64.94) | 0.49  (0.21–1.18) | 0.46  (0.17–1.24) | 0.60  (0.25–1.45) | 0.53  (0.11–2.45) |
|  |  | LMWH | 5181 | 163 | 2975.07 | 54.79  (46.70–63.88) | 1.00 (Ref) | 1.00 (Ref) | 1.00 (Ref) | 1.00 (Ref) |
|  | Overall | RVX | 283 | 5 | 184.69 | 27.07  (8.79–63.18) | 0.49  (0.20–1.18) | 0.45  (0.17–1.21) | 0.60  (0.25–1.45) | 0.51  (0.11–2.32) |
|  |  | LMWH | 5181 | 166 | 3147.91 | 52.73  (45.02–61.39) | 1.00 (Ref) | 1.00 (Ref) | 1.00 (Ref) | 1.00 (Ref) |
| All-cause  mortality | 0–3 | RVX | 283 | 10 | 61.15 | 163.5  (78.42–300.8) | 0.17  (0.09–0.32) | 0.36  (0.19–0.69) | Competing risk | |
|  |  | LMWH | 5181 | 929 | 1083.95 | 857.1  (802.8–914.0) | 1.00 (Ref) | 1.00 (Ref) |  |  |
|  | 0–6 | RVX | 283 | 14 | 108.65 | 128.9  (70.45–216.2) | 0.15  (0.09–0.26) | 0.29  (0.17–0.50) |  |  |
|  |  | LMWH | 5181 | 1421 | 1823.91 | 779.1  (739.1–820.7) | 1.00 (Ref) | 1.00 (Ref) |  |  |
|  | 0–12 | RVX | 283 | 18 | 154.77 | 116.3  (68.93–183.8) | 0.15  (0.10–0.25) | 0.27  (0.17–0.45) |  |  |
|  |  | LMWH | 5181 | 1811 | 2623.83 | 690.2  (658.8–722.7) | 1.00 (Ref) | 1.00 (Ref) |  |  |
|  | 0–24 | RVX | 283 | 19 | 180.69 | 105.2  (63.31–164.2) | 0.15  (0.09–0.23) | 0.25  (0.15–0.40) |  |  |
|  |  | LMWH | 5181 | 2027 | 3037.97 | 667.2  (638.5–696.9) | 1.00 (Ref) | 1.00 (Ref) |  |  |
|  | Overall | RVX | 283 | 20 | 185.71 | 107.7  (65.78–166.3) | 0.15  (0.10–0.23) | 0.26  (0.16–0.41) |  |  |
|  |  | LMWH | 5181 | 2109 | 3223.79 | 654.2  (626.6–682.7) | 1.00 (Ref) | 1.00 (Ref) |  |  |

CI, confidence interval; HR, hazard ratio; IR, incidence rate; LMWH, low molecular weight heparin; RVX, rivaroxaban; VTE, venous thromboembolism. ^a^Adjusted for sex and age.

**Table S5.** Incidence rates, unweighted and weighted hazard ratios from Cox regression and unweighted and weighted subhazards from Fine–Gray regression for recurrent VTE and major bleeding; DOACs (N = 566) vs LMWH (N = 5181) under ITT exposure definition

| **Outcome** | **Months follow-up** | **Group** | **Events**  **(N)** | **Person-years** | **IR (CI) per 1000 PY** | **Unweighted**  **HR* (CI)** | **Weighted**  **sub-HR (CI)** | **Unweighted**  **sub-HR^a^ (CI)** | **Weighted**  **sub-HR (CI)** |
| --- | --- | --- | --- | --- | --- | --- | --- | --- | --- |
| **Recurrent VTE** | 0-3 | DOAC | 25 | 127 | 196.6  (127.2-290.2) | 0.98  (0.65-1.49) | 0.96  (0.59-1.56) | 1.00  (0.66-1.52) | 0.96  (0.49-1.89) |
|  |  | LMWH | 230 | 1121 | 205.2  (179.5-233.5) | 1.00 (Ref) | 1.00 (Ref) | 1.00 (Ref) | 1.00 (Ref) |
|  | 0-6 | DOAC | 27 | 246 | 109.8  (72.34-159.7) | 0.90  (0.61-1.35) | 0.89  (0.56-1.42) | 0.95  (0.64-1.41) | 0.91  (0.48-1.72) |
|  |  | LMWH | 262 | 2043 | 128.3  (113.2-144.8) | 1.00 (Ref) | 1.00 (Ref) | 1.00 (Ref) | 1.00 (Ref) |
|  | 0-12 | DOAC | 35 | 466 | 75.18  (52.36-104.6) | 0.88  (0.62-1.25) | 0.91  (0.61-1.35) | 0.98  (0.69-1.39) | 0.95  (0.54-1.65) |
|  |  | LMWH | 328 | 3583 | 91.55  (81.91-102.0) | 1.00 (Ref) | 1.00 (Ref) | 1.00 (Ref) | 1.00 (Ref) |
|  | 0-24 | DOAC | 45 | 818 | 55.00  (40.12-73.59) | 0.89  (0.65-1.22) | 0.90  (0.63-1.29) | 1.06  (0.78-1.44) | 0.97  (0.58-1.60) |
|  |  | LMWH | 393 | 5896 | 66.66  (60.23-73.59) | 1.00 (Ref) | 1.00 (Ref) | 1.00 (Ref) | 1.00 (Ref) |
|  | Overall | DOAC | 56 | 1396 | 40.12  (30.31-52.10) | 0.92  (0.70-1.22) | 0.94  (0.68-1.29) | 1.15  (0.87-1.52) | 1.05  (0.66-1.66) |
|  |  | LMWH | 473 | 10524 | 44.95  (40.99-49.19) | 1.00 (Ref) | 1.00 (Ref) | 1.00 (Ref) | 1.00 (Ref) |
| **Major bleeding** | 0-3 | DOAC | 5 | 131 | 38.04  (12.35-88.77) | 0.45  (0.18-1.10) | 0.45  (0.17-1.17) | 0.48  (0.19-1.17) | 0.45  (0.13-1.54) |
|  |  | LMWH | 93 | 1150 | 80.87  (65.27-99.07) | 1.00 (Ref) | 1.00 (Ref) | 1.00 (Ref) | 1.00 (Ref) |
|  | 0-6 | DOAC | 8 | 254 | 31.48  (13.59-62.04) | 0.44  (0.22-0.90) | 0.48  (0.22-1.05) | 0.49  (0.24-1.01) | 0.50  (0.18-1.38) |
|  |  | LMWH | 141 | 2096 | 67.26  (56.62-79.32) | 1.00 (Ref) | 1.00 (Ref) | 1.00 (Ref) | 1.00 (Ref) |
|  | 0-12 | DOAC | 14 | 483 | 28.96  (15.83-48.59) | 0.59  (0.34-1.01) | 0.65  (0.36-1.17) | 0.69  (0.40-1.20) | 0.69  (0.31-1.54) |
|  |  | LMWH | 181 | 3682 | 49.16  (42.26-56.86) | 1.00 (Ref) | 1.00 (Ref) | 1.00 (Ref) | 1.00 (Ref) |
|  | 0-24 | DOAC | 22 | 852 | 25.81  (16.18-39.08) | 0.72  (0.47-1.12) | 0.77  (0.47-1.26) | 0.91  (0.59-1.41) | 0.86  (0.43-1.71) |
|  |  | LMWH | 218 | 6063 | 35.96  (31.34-41.06) | 1.00 (Ref) | 1.00 (Ref) | 1.00 (Ref) | 1.00 (Ref) |
|  | Overall | DOAC | 31 | 1464 | 21.18  (14.39-30.06) | 0.82  (0.57-1.18) | 0.86  (0.56-1.31) | 1.08  (0.75-1.56) | 1.00  (0.54-1.84) |
|  |  | LMWH | 273 | 10918 | 25.00  (22.13-28.15) | 1.00 (Ref) | 1.00 (Ref) | 1.00 (Ref) | 1.00 (Ref) |
| **All-cause mortality** | 0-3 | DOAC | 39 | 132 | 295.4  (210.1-403.9) | 0.32  (0.23-0.44) | 0.65  (0.46-0.91) | Competing risk | |
|  |  | LMWH | 955 | 1161 | 822.7  (771.3-876.5) | 1.00 (Ref) | 1.00 (Ref) |  |  |
|  | 0-6 | DOAC | 65 | 257 | 253.4  (195.6-323.0) | 0.32  (0.25-0.41) | 0.60  (0.46-0.79) |  |  |
|  |  | LMWH | 1522 | 2127 | 715.4  (679.9-752.3) | 1.00 (Ref) | 1.00 (Ref) |  |  |
|  | 0-12 | DOAC | 94 | 490 | 191.9  (155.0-234.8) | 0.32  (0.26-0.39) | 0.57  (0.45-0.71) |  |  |
|  |  | LMWH | 2125 | 3757 | 565.6  (541.8-590.2) | 1.00 (Ref) | 1.00 (Ref) |  |  |
|  | 0-24 | DOAC | 127 | 869 | 146.2  (121.9-174.0) | 0.33  (0.28-0.39) | 0.57  (0.47-0.68) |  |  |
|  |  | LMWH | 2628 | 6220 | 422.5  (406.5-439.0) | 1.00 (Ref) | 1.00 (Ref) |  |  |
|  | Overall | DOAC | 153 | 1502 | 101.9  (86.38-119.4) | 0.34  (0.29-0.40) | 0.56  (0.47-0.66) |  |  |
|  |  | LMWH | 3084 | 11309 | 272.7  (263.2-282.5) | 1.00 (Ref) | 1.00 (Ref) |  |  |

CI, confidence interval; HR, hazard ratio; IR, incidence rate; LMWH, low molecular weight heparin; RVX, rivaroxaban; VTE, venous thromboembolism. ^a^Adjusted for sex and age.

Supplementary figures

**Supplementary Fig. S1** Baseline characteristics before and after PS weighting under ITT exposure; rivaroxaban vs LMWH


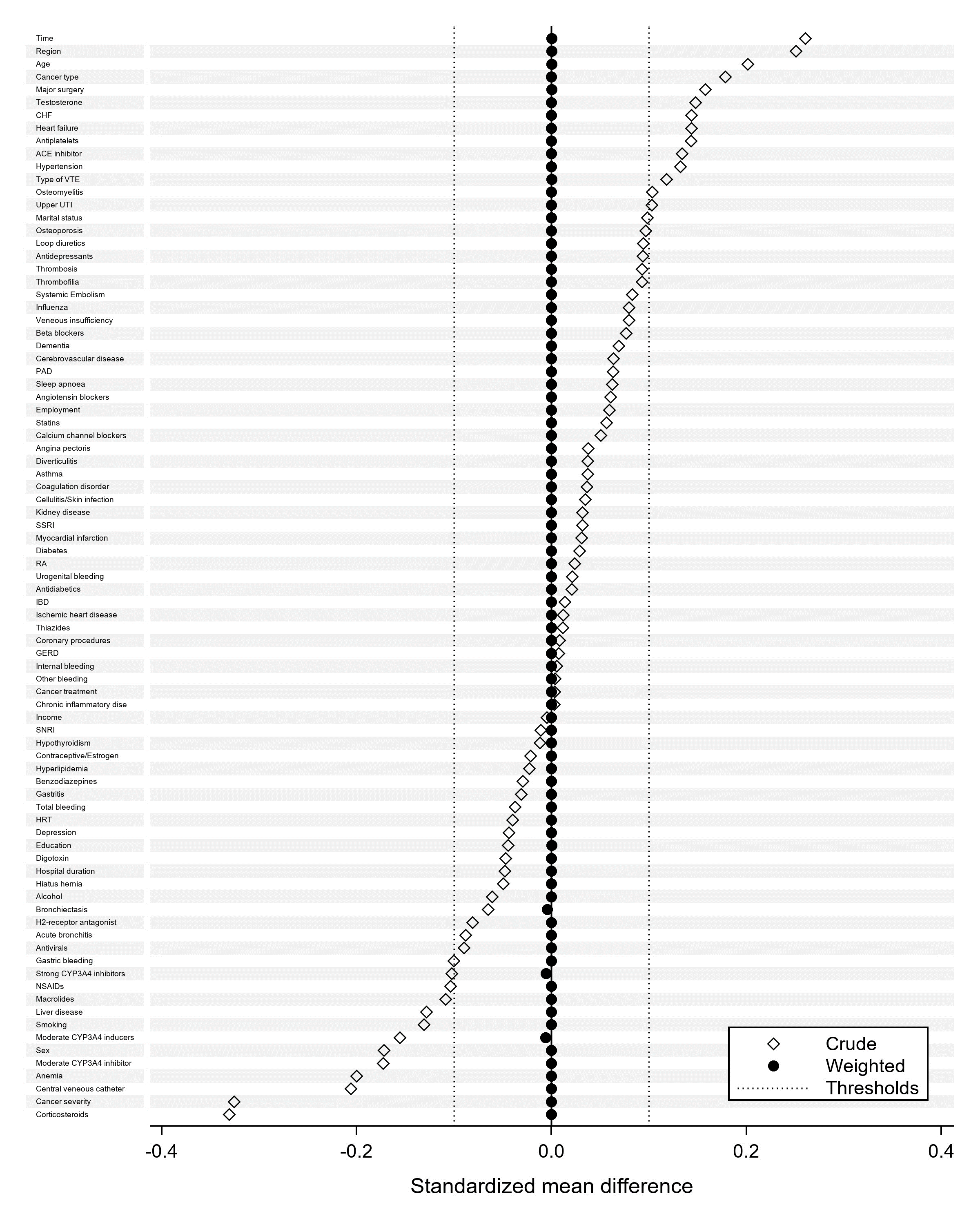


Graph created using SAS software, Version 9.4 of the SAS System for Windows. Copyright © 2013 SAS Institute Inc

Supplementary reference

1. J Montomoli, R Erichsen, S Antonsen, T Nilsson and HT Sorensen (2015) Impact of preoperative serum albumin on 30-day mortality following surgery for colorectal cancer: a population-based cohort study. BMJ Open Gastroenterol 2:e000047. <https://doi.org/10.1136/bmjgast-2015-000047>
